# Le doigt pelvien: une anomalie osseuse rare

**DOI:** 10.11604/pamj.2016.25.1.10373

**Published:** 2016-08-31

**Authors:** Imad Ghozlani, Abdennasser El Kharras

**Affiliations:** 1 Service de Rhumatologie, 1^er^ Centre Médico Chirurgical, Agadir, Maroc; 2 Service d’Imagerie Médicale, 1^er^ Centre Médico Chirurgical, Agadir, Maroc

**Keywords:** Doigt pelvien, anomalie rare, protubérance osseuse, pelvic digit, rare abnormality, bone protuberance

## Image en médecine

Le doigt pelvien est une anomalie anatomique rare caractérisée par le développement d’une structure osseuse dans les tissus mous adjacents au pelvis. Son origine est encore inconnue, mais la théorie suggère une anomalie apparaissant au stade mésenchymateux de la croissance osseuse au cours des six premières semaines d’embryogenèse. Nous présentons le cas d’un patient de 40 ans, grand sportif et qui consultait pour une douleur mécanique du pli inguinal droit survenue lors d’une séance d’entrainement. L’examen clinique trouvait une hanche droite douloureuse à la mobilisation mais sans limitation. Le reste de l’examen somatique était sans anomalies. La radiographie du bassin face a mis en évidence une protubérance osseuse en forme de doigt s’articulant avec l’angle supéro externe du toit du cotyle droit (A). La tomodensitométrie a montré le doigt pelvien, avec une corticale bien différenciée, s’articulant avec le toit du cotyle droit (B) et s’étendant en direction antéro-inférieure (C). Cette anomalie peut survenir à n’importe quel niveau des os pelviens, et peut même être localisée entièrement à l’intérieur des tissus mous de la paroi abdominale. La tomodensitométrie est utile dans ces cas car elle permet d’établir un diagnostic différentiel (tumeurs osseuses, ostéochondrome, myosite ossifiante et des fracture-avulsions du bassin). Occasionnellement, le doigt pelvien peut être bilatéral ou multiple. Il est généralement asymptomatique et de découverte fortuite. Il peut parfois être une source de douleur, de difficulté ou d’handicap fonctionnel à cause de sa proximité avec l’articulation. Dans ce cas, une excision chirurgicale peut être nécessaire.

**Figure 1:**
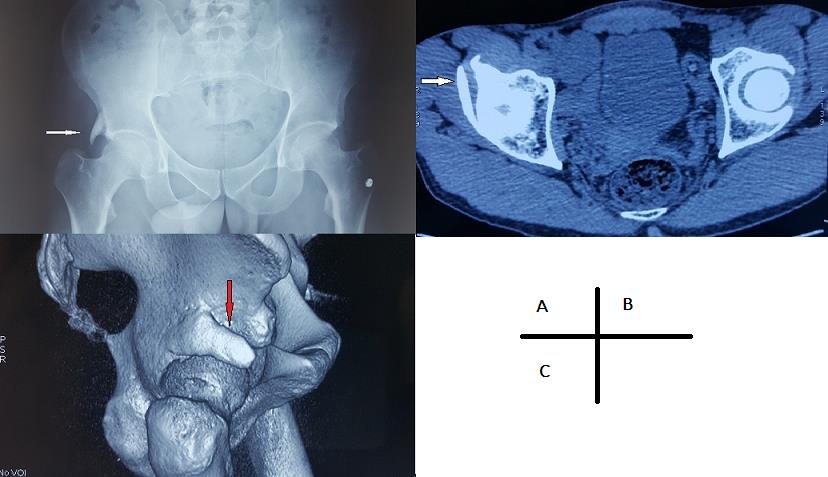
A) radiographie standard du bassin face montrant une protubérance osseuse en forme de doigt; B) tomodensitométrie en coupe axiale montrant le doigt pelvien s’articulant avec le toit du cotyle droit; C) tomodensitométrie avec reconstruction 3D montrant le doigt pelvien s’étendant en direction antéro-inférieure

